# Chronic Liver-Specific LRP1 Silencing Drives Progressive Brain Amyloidosis, Neuroinflammation, and Behavioral Deficits in APP/PS1 Mice

**DOI:** 10.21203/rs.3.rs-10843451/v1

**Published:** 2026-09-03

**Authors:** Devaraj V. Chandrashekar, G. Chuli Roules, Urvashi Panchal, Nataraj Jagadeesan, Cody Newhart, Josephine Chu, Brian Carson, Sanda Win, Tin A. Than, Neil Kaplowitz, Jerome Garcia, Derick Han, Rachita K. Sumbria

**Affiliations:** Chapman University; Chapman University; Chapman University; Chapman University; Chapman University; Keck Graduate Institute; Keck Graduate Institute; University of Southern California; University of Southern California; University of Southern California; University of La Verne; Keck Graduate Institute; Chapman University

**Keywords:** Aβ, APP/PS1 mice, LRP1, liver, APP, miR-LRP1, thyroxine binding globulin, Alzheimer’s disease, liver-brain axis

## Abstract

Liver dysfunction is increasingly implicated in Alzheimer’s disease (AD) pathogenesis, yet the underlying mechanisms remain poorly understood. Hepatic low-density lipoprotein receptor-related protein 1 (LRP1), the principal receptor mediating peripheral amyloid-beta (Aβ) clearance, is downregulated in chronic liver diseases. Using an AAV8-TBG hepato-specific silencing strategy in APP/PS1 mice, we previously demonstrated that 12 weeks of hepatic LRP1 silencing elevated soluble brain Aβ and locomotor hyperactivity without affecting insoluble Aβ, neuroinflammation, or cognition. Here, we extended silencing to 28 weeks to model hepatic LRP1 reduction with chronic liver dysfunction and assessed whether sustained hepatic LRP1 loss drives a more severe AD phenotype. Hepatic LRP1 was reduced by 70% with no off-target effects on brain or kidney LRP1, liver histology, or systemic inflammation, confirming liver specificity and safety. Prolonged silencing produced a time-dependent progression of brain Aβ accumulation, shifting from soluble to insoluble Aβ species from 12 to 28 weeks of hepatic LRP1 silencing. Elevated plasma Aβ42 was strongly and positively correlated with insoluble brain Aβ42 (r = 0.65, p < 0.05), supporting a liver-plasma-brain axis in modulating brain Aβ. Sustained hepatic LRP1 silencing was accompanied by reactive astrogliosis (p < 0.01), microglial transition to a disease-associated state (p < 0.05), increased brain ApoE (p < 0.05), and worsened locomotor hyperactivity (p < 0.05) and spatial memory impairment (p = 0.056). Mechanistically, hepatic LRP1 silencing transiently increased hepatic APP expression (p < 0.01) and elevated plasma ApoB48 (p < 0.05), suggesting dual contributions of reduced Aβ clearance and increased peripheral Aβ production. Absence of compensatory changes in other hepatic Aβ transporters confirms that the observed AD phenotypes are driven by alterations in hepatic LRP1. These findings underscore hepatic LRP1 as a critical regulator of Aβ pathology. Future work will further highlight the potential of hepatic LRP1 as a therapeutic target and perhaps as a biomarker for AD risk associated with liver disease.

## 1. Introduction

Low-density lipoprotein receptor-related protein 1 (LRP1) is a multifunctional endocytic receptor, highly expressed in the liver and brain that has received considerable attention for its role in Alzheimer’s disease (AD) [17, 32, 44]. LRP1 binds and internalizes over 40 ligands, including apolipoprotein E (ApoE) and amyloid-β (Aβ), both of which are highly relevant to AD [34]. As the predominant peripheral clearance receptor for circulating Aβ [45], hepatic LRP1 facilitates receptor-mediated endocytosis and lysosomal catabolism of Aβ, while in the brain, LRP1 plays an important role in Aβ clearance within the parenchyma and efflux across the blood-brain barrier (BBB) [24, 34, 40].

Although AD is largely viewed as a brain disease, systemic factors are now increasingly implicated in AD. Notably, clinical studies have linked liver injury markers to AD biomarkers, cognitive deficits following liver transplants have been linked to increased serum Aβ levels, and liver dysfunction increases AD pathology, highlighting the liver-brain axis in AD [3, 27, 31, 37]. A key player underlying liver-brain crosstalk in AD appears to be hepatic LRP1 [44]. Dysfunction or downregulation of hepatic LRP1 has been reported in liver disease, including non-alcoholic fatty liver disease and hepatocellular carcinoma [16, 19]. Consistent with this, we found reduced hepatic LRP1 expression in steatotic (fatty) liver associated with obesity and alcohol intake in rodent models [4, 12], and hepatic LRP1 deficiency aggravated high-fat diet-induced liver steatosis [9]. Given the central role of hepatic LRP1 in peripheral Aβ clearance [45], dysfunction or downregulation of hepatic LRP1, as seen in liver disease [16, 19], may impair peripheral Aβ clearance and drive Aβ accumulation in the brain. This may be a mechanistically important pathway in AD pathogenesis and progression in conditions of liver disease.

Three lines of evidence support the role of hepatic LRP1 in AD pathogenesis. First, individuals carrying the rs1799986 T allele have a lower risk of AD and reduced plasma Aβ levels, and higher LRP1 levels with the T allele are associated with this protective effect in AD [25, 39]. Second, experimental reduction of hepatic LRP1 markedly impairs the liver’s capacity to clear circulating Aβ [8, 45]. Third, we previously showed that short-term (12 weeks) hepato-specific LRP1 silencing in four-month-old APP/PS1 mice increased soluble brain Aβ and locomotor hyperactivity, without affecting insoluble Aβ burden, neuroinflammation, or cognitive function [4]. Since chronic liver injury is likely associated with sustained hepatic LRP1 dysfunction, our goals were twofold: to determine whether prolonged hepatic LRP1 silencing would unmask a more severe AD pathological phenotype, and to establish the temporal profile of Aβ accumulation in the brain with prolonged hepatic LRP1 silencing. We therefore hypothesized that increasing the duration of hepatic LRP1 silencing from 12 to 28 weeks would shift the Aβ equilibrium toward insoluble Aβ species and produce measurable cognitive deficits, more closely recapitulating the sustained hepatic LRP1 impairment observed in clinical liver disease [16, 19].

To investigate the impact of prolonged hepatic LRP1 loss on AD pathology, four-month-old male APP/PS1 mice received liver-targeted LRP1 silencing via intravenous (IV) administration of an AAV8 vector encoding LRP1-specific microRNA under the transcriptional control of the hepatocyte-selective thyroxine-binding globulin (TBG) promoter (AAV8 miR-LRP1). APP/PS1 control mice received a control AAV8 construct (AAV8 control). Following seven months of hepatic LRP1 silencing, behavior tests were performed to evaluate spatial reference memory and exploratory locomotor activity using the Y-maze and open-field tests, respectively. At the study endpoint, brain, liver, and plasma were collected and analyzed for LRP1 expression, Aβ accumulation, neuroinflammation, and lipoprotein profiles, including ApoE and apolipoprotein B (ApoB), using immunoassays.

## 2. Materials and methods

### 2.1. Animal studies

The article follows the ARRIVE guidelines 2.0 for reporting animal research.

#### 2.1.1. Liver-specific silencing of LRP1 in APP/PS1 mice.

Male double-transgenic APP/PS1 mice (APPswe/PSEN1dE9; stock #034829-JAX) [22] were obtained from the Jackson Laboratory (Bar Harbor, ME) and maintained under controlled temperature and humidity conditions with unrestricted access to food and water. All experimental procedures were reviewed and approved by the Chapman University Institutional Animal Care and Use Committee (IACUC; protocol #2020 – 1170). Prior to treatment, animals were individually housed and stratified by body weight into two experimental groups: AAV8 control group (n = 6) receiving IV administration of 5×10^1^^2^ gene copies (GC)/mouse of AAV8.TBG.PI.eGFP.miR(3xLacZ).WPRE.bGH, and the AAV8 miR-LRP1 group (n = 7) receiving 5×10^1^^2^ GC/mouse of AAV8.TBG.PI.eGFP.miR(Mouse Lrp1×3).WPRE.bGH (V7914R) [4]. Both vectors were manufactured by the Penn Vector Core (Philadelphia, PA) and delivered via a tail vein injection at four months of age. Hepatocyte-selective LRP1 silencing in the AAV8 miR-LRP1 group was achieved through transcriptional regulation by the liver-specific TBG promoter [28]. Mice were sacrificed 28 weeks post-injection at 11 months of age. Terminal blood collection was performed before anesthesia with Euthasol (150 mg/kg, IP), followed by cardiac perfusion with ice-cold phosphate-buffered saline (PBS). Brain, liver, and kidneys were harvested; right hemispheres were immersion-fixed in 4% paraformaldehyde (PFA) for immunohistochemical analyses, and left hemispheres were snap-frozen in liquid nitrogen for Western blot and ELISA. The excised liver was weighed, a part was fixed in 4% PFA, and the remainder was stored at − 80°C for further analysis.

### 2.2. Brain sectioning and immunostaining

Right brain hemispheres, previously fixed and cryoprotected, were embedded in Tissue-Tek OCT compound (Fisher Scientific, MA, USA) and sectioned sagittally at 20 μm thickness using a Leica CM1850 cryostat (Micron Instruments, CA, USA). Sections were collected and stored in PBS supplemented with 0.01% sodium azide at 4°C until immunostaining. Three sections per mouse spaced 600 μm apart were selected for subsequent immunohistochemical analysis.

### 2.3 6E10 immunofluorescence

Aβ deposition was visualized using an Alexa Fluor 488 mouse monoclonal antibody targeting human Aβ (hAβ) residues 1–16 (clone 6E10, 1:1000; BioLegend, CA, USA). Free-floating sagittal sections were rinsed in 1× PBS, subjected to antigen retrieval in 70% formic acid for 10 minutes at room temperature, and blocked in 0.5% bovine serum albumin (BSA) with 0.3% Triton X-100 in PBS for 1 hour. Sections were incubated overnight at 4°C with the 6E10 antibody, washed, mounted onto glass slides, coverslipped with Vectamount aqueous mounting medium (Vector Laboratories, CA, USA), and stored at 4°C. Full section images were acquired at 20× magnification using a BZ-X710 Keyence fluorescence microscope (Keyence, Itasca, IL, USA) and digitally stitched. Aβ-positive count (normalized to tissue area), area (% of brain section area), and size (μm^2^; normalized to tissue area) were quantified using NIH ImageJ (version 1.53e; National Institutes of Health, Bethesda, MD, USA) by two blinded independent observers [38].

### 2.4 Thioflavin-S staining

To assess mature Aβ plaque deposition, brain sections from control and LRP1-silenced groups were processed for Thioflavin-S staining. Free-floating sections were first washed in 1× PBS and mounted onto positively charged microscope slides. Once air-dried, slides were rinsed briefly with distilled water to eliminate residual salts and subsequently dehydrated through graded ethanol solutions (70% and 80%, 1 minute each). Sections were then incubated in a freshly prepared, filtered 1% Thioflavin-S solution in 80% ethanol for 15 minutes at room temperature. Following staining, the sections were again submerged in 70% and 80% ethanol solution for 1 minute each, washed with distilled water, and allowed to air-dry. Sections were cover slipped using Vectamount aqueous mounting medium and imaged using a BZ-X710 Keyence fluorescence microscope (Keyence, Itasca, IL, USA) at 20x magnification and stitched together using the stitching tool to capture the entire brain section within a single field of view. Images were analyzed using NIH ImageJ to quantify Thioflavin-S-positive counts (normalized to tissue area), area (expressed as a % of brain section area), and stain size (μm^2^; normalized to tissue area) by two readers blinded to the experimental groups, as described previously [38].

### 2.5 TREM2, Iba1, and GFAP-triple immunofluorescence staining

Triggering receptor expressed on myeloid cell 2 (TREM2) is an activating cell-surface receptor expressed on dendritic cells, osteoclasts, and microglia in the brain. In microglia, TREM2 signaling promotes cell activation, phagocytosis, and overall neuroprotection; however, its dysregulation can also contribute to neurodegenerative processes [47]. Ionized calcium-binding adaptor molecule-1 (Iba1) is a commonly used marker for microglia and macrophages and is upregulated upon their activation [20]. Glial fibrillary acidic protein (GFAP) is an intermediate filament protein expressed by adult astrocytes, and is upregulated in reactive astrocytes [18]. For TREM2, Iba1, and GFAP immunostaining, free-floating brain sections from control and LRP1-silenced groups were washed three times in 1× PBS for 5 minutes each, followed by antigen retrieval in sodium citrate buffer (10 mM sodium citrate, 0.05% Tween-20, pH 6.0) for 15 minutes. Tissues were then blocked for 60 minutes at room temperature using a blocking solution containing 0.5% BSA and 0.3% Triton X-100 in PBS. Sections were incubated overnight at 4°C with primary antibodies against Iba1 (rabbit, Wako #019–19741, 1:1000), TREM2 (polyclonal sheep IgG, R&D Systems #AF1729, 1:250), and GFAP (mouse monoclonal GA5, Alexa Fluor^™^ 488, Invitrogen #53-9892-82, 1:500). All subsequent steps were performed in the dark. The following day, sections were washed three times in 1× PBS and incubated for 2 hours at room temperature with corresponding secondary antibodies, including Alexa Fluor 594 donkey anti-rabbit IgG for Iba1 and Alexa Fluor 647 donkey anti-sheep IgG (H + L) for TREM2, prepared in 0.5% BSA with 0.3% Triton X-100 in PBS. After incubation, sections were washed in PBS, briefly rinsed with distilled water to remove residual salts, mounted onto positively charged glass slides, and coverslipped using Vectamount aqueous mounting medium after complete air-drying. Three brain sections were imaged per mouse, and for each brain section, two regions each in the cerebral cortex and hippocampus, and one region each in the amygdala and entorhinal cortex, were imaged using a Nikon Ti-E Confocal Microscope (Nikon Instruments Inc., Melville, NY, USA) at 40x magnification. Stain-positive area was quantified using predefined thresholds in ImageJ, as described previously [6].

### 2.6 Brain and liver tissue homogenization

Frozen left-hemibrains were pulverized under dry ice, and a portion was homogenized in Tris-buffered saline (TBS; 1:10, w/v) containing 50 mM Tris-HCl (pH 7.6), 150 mM NaCl, 5 mM EDTA, 2 mM 1,10-phenanthroline, and Roche Complete EDTA-free Mini protease inhibitor. The homogenates were centrifuged at 100,000 ×g for 1 h at 4°C, and the resulting supernatants (TBS-soluble fractions) were collected, aliquoted to avoid repeated freeze–thaw cycles, and stored at − 80°C until further analysis. The pellets were resuspended in ten volumes of homogenization buffer containing 5 M guanidine-HCl and 0.05 M Tris (pH 8.0) and re-homogenized. Samples were incubated on a rotor for 2 h at room temperature, followed by centrifugation at 20,800 ×g for 15 min. The supernatants (Gu-HCl-soluble fractions) were collected, aliquoted, and stored at − 80°C until analysis.

A separate portion of the pulverized brain tissue and liver was homogenized in 15 volumes of radioimmunoprecipitation assay (RIPA) buffer containing Roche Complete EDTA-free Mini protease inhibitor and allowed to shake on a rotor for 1 h at 4°C. The brain and liver homogenates were then centrifuged at 12,000 ×g for 20 min at 4°C, and the supernatants (RIPA fractions) were collected and stored at − 80°C for further use. Protein concentrations in all brain fractions (TBS-, Gu-HCl-soluble, and RIPA), and RIPA liver fractions were determined using the bicinchoninic acid (BCA) assay (Pierce Chemical Co., Rockford, IL, USA).

### 2.7 Quantification of Aβ by ELISA

Human and mouse Aβ42 (hAβ42 and mAβ42, respectively) were quantified in the TBS-soluble and Gu-HCl-soluble brain fractions, as well as in terminal plasma samples, using commercially available ELISA kits specific for human (#KHB3441) and mouse (#KMB3441) Aβ42 (Thermo Fisher Scientific, CA, USA), following the manufacturer’s instructions.

### 2.8. Liver profile

To evaluate hepatotoxicity, serum was isolated from terminal blood samples and analyzed using the Vetscan VS2 Chemistry Analyzer (Abaxis, Zoetis, NJ, USA) with mammalian liver profile rotors (Zoetis, NJ, USA). Serum samples were diluted 1:1 with normal saline prior to loading onto the rotors. The following liver injury markers were quantified: alanine aminotransferase (ALT), alkaline phosphatase (ALP), albumin, blood urea nitrogen (BUN), and cholesterol.

### 2.9 Liver histology

To assess hepatic histology, following fixation in 4% PFA and storage in 70% ethanol, liver tissues were paraffin-embedded and cut into serial sections of 5 μm thickness. H&E staining was carried out through the Research Services Core of the Department of Pathology and Laboratory Medicine at UCI Medical Center. Stained sections were visualized under a light microscope, and representative images were captured at 200x magnification.

### 2.10 Western blot analysis

RIPA-soluble tissue fractions (~ 25 μg protein/lane) were processed in 4x-lamelli buffer and separated by SDS-polyacrylamide gel electrophoresis using a precast 4–20% MP TGX gels (Bio-Rad, Hercules, CA, USA) at a constant voltage of 100 V for 60 min. Proteins from the gel were transferred onto a polyvinylidene fluoride (PVDF) membrane (0.45 μm) using Tank Transfer System (Bio-Rad, Hercules, CA, USA), followed by blocking of the PVDF membrane by incubating in 3% milk for one hour at room temperature. After blocking, the membranes were incubated overnight at 4°C with their respective primary antibodies diluted in 3% milk prepared in 1x TBS. The primary antibodies used were: mouse/human APP (1:1000, #76600, Cell Signaling Technology, Danvers, MA, USA), LRP1 (1:1000, #64099, Cell Signaling Technology), apolipoprotein E (ApoE; 1:1000, #49285, Cell Signaling Technology), low-density lipoprotein receptor-related protein 6 (LRP6; 1:1000, #3395, Cell Signaling Technology), presenilin-2 (PSEN2; 1:1000, #9979, Cell Signaling Technology), multidrug resistance protein 1 (MDR1; 1:1000, #13978, Cell Signaling Technology), receptor for advanced glycation end products (RAGE; 1:1000, #55222, Cell Signaling Technology), and β-actin as a loading control (1:1000, #sc-47778, Santa Cruz Biotechnology, Santa Cruz, CA, USA; or 1:1000, #4970, Cell Signaling Technology). After washing, the blots were incubated with their respective HRP-conjugated IgG anti-rabbit (1:1000, #7074, Cell Signaling, Danvers, MA, USA), or HRP-conjugated anti-mouse IgG kappa (1:1000, #sc-516102, Santa Cruz, CA, USA), secondary antibodies for one hour at room temperature followed by washing before visualization of bands with the Bio-Rad Chemi Doc Imager system (Bio-Rad, Hercules, CA, USA). NIH ImageJ was used to quantify the intensity of the Western blot bands, and protein expression was normalized to the β-actin protein expression. Data was represented as % of the control-treated group.

### 2.11 Behavior study

#### 2.11.1. Open field test

Exploratory locomotor behavior was assessed in 11-month-old LRP1-silenced and control APP/PS1 mice using the open field test, as previously described [4]. Testing was conducted in a 90 × 90 × 40 cm open-field arena equipped with dividers to simultaneously accommodate four animals (Panlab, Harvard Apparatus, MA, USA). Prior to testing, all animals were transferred to the testing room and allowed to acclimate for 30 minutes to minimize stress-related confounds. Each mouse was then individually introduced into one of the four arena compartments, and spontaneous locomotor activity was recorded for a duration of 5 minutes using a digital camera system. To prevent olfactory bias between trials, the arena was thoroughly wiped down with 70% ethanol between each animal. Recorded video files were analyzed using SMART Video Multi-Arena Tracking Software (Panlab, Harvard Apparatus, MA, USA) to determine mean locomotor speed and total distance traveled for each animal.

#### 2.11.2. Y-Maze test

Spatial reference memory was assessed in APP/PS1 mice following hepatic LRP1 silencing using the Y-maze test, as previously described [4]. The apparatus consisted of three symmetrical radial arms (30 cm length × 6 cm width × 15 cm height). Testing was conducted in two sequential phases: a training phase and a testing phase. During the training phase, the novel arm was blocked, and each mouse was placed at the entry of the start arm to freely explore the start and familiar arms for 8 minutes. Following a 30-minute interval, the blocked arm was opened, and the mouse was reintroduced to the apparatus and allowed to explore all three arms for an additional 8 minutes. Mouse movement was recorded and analyzed using SMART Video Tracking Software (Panlab, Harvard Apparatus). The discrimination index, defined as the ratio of time spent exploring the novel arm relative to the novel, familiar, and start arms, was calculated as a measure of spatial recognition memory.

#### 2.11.3. Nesting behavior test

Nest-building behavior was assessed as an index of general health and well-being in both LRP1-silenced and control APP/PS1 mice. Each animal was individually housed in a cage containing a single pre-weighed Nestlet (~ 2.5 g; Newco Specialty). After 24 hours, nesting quality was independently evaluated by two observers blinded to the experimental group assignments, using a standardized scoring scale as previously described [5].

### 2.12 Cytokine measurements

Terminal plasma samples were diluted twofold in assay diluent buffer and analyzed using the V-PLEX Proinflammatory Panel 1 multiplex assay according to the manufacturer’s instructions (MesoScale Discovery, Gaithersburg, MD, USA). Plates were read on a SECTOR Imager MSD QuickPlex SQ 120 plate reader (MesoScale Discovery, Gaithersburg, MD, USA), and data were processed and analyzed using the MSD Discovery Workbench software.

### 2.13 Statistical analysis

All data are reported as mean ± standard error of the mean (SEM). A priori sample size estimation was performed using G*Power to compare two independent groups (control AAV8 and AAV8 miR-LRP-1 mice). Based on a minimum expected difference in mean Aβ measurements of 50% derived from previous work [4], a standard deviation of 25%, and a desired power of 80%, a sample size of six mice per group was determined to be sufficient. Prior to analysis, outliers were identified using Grubbs’ test. For comparisons between two groups, parametric data were analyzed using an unpaired Student’s t-test, with Welch’s correction applied where variances were unequal. Non-parametric data were assessed using the Mann-Whitney U test. For immunostaining analyses, where multiple images were acquired per mouse, a linear mixed-effects (LME) model was implemented in MATLAB to account for the nested data structure, with treatment group (control vs. miR-LRP1) included as a fixed effect and individual mouse as a random effect [53]. Statistical significance was defined as p ≤ 0.05.

## 3. Results

### 3.1. Targeted silencing of LRP1 in the liver using AAV8 driven by the TBG promoter in aged APP/PS1 mice

The experimental design for sustained hepatic LRP1 silencing, achieved through a LRP1-specific microRNA packaged into an AAV8 vector under the control of the liver-specific TBG promoter [4], in APP/PS1 mice, is outlined in Fig. 1A. Western blot data confirm the liver-specific LRP1 silencing (70% reduction in hepatic LRP1) (Fig. 1B), with no change in LRP1 expression in the brain (Fig. 1B) or kidney (Supplemental Figure. 1A), consistent with our previous work showing no change in LRP1 expression in other organs [4]. Hepato-specific LRP1 silencing did not cause liver injury, as indicated by no significant changes in hepatocyte histology (Fig. 1C) and ALT levels (Fig. 1D). To study the general peripheral effects of hepatic LRP1 silencing, we performed a multiplex cytokine assay on the terminal plasma samples and did not find any significant changes in the AAV8 miR-LRP1-injected APP/PS1 mice compared with the control AAV8-injected APP/PS1 mice (Fig. 1E). Plasma cholesterol, albumin, and ALP levels were slightly but significantly reduced with hepatic LRP1 silencing, with no change in the BUN (Supplemental Figure. 1B-E).

**(A)** Schematic of the experimental design created using BioRender. Male APP/PS1 mice were injected with either AAV8 Control or AAV8 miR-LRP1 at 4 months of age and aged to 11 months. Animals underwent a battery of behavioral assessments, including nesting, open-field, and Y-maze tests. At endpoint, brain, plasma, and liver tissues were collected to assess amyloid-beta (Aβ) levels and LRP1 expression. **(B)** Representative western blots of LRP1 protein expression in liver (top, red panel) and brain (middle, blue panel) from AAV8 Control and AAV8 miR-LRP1 mice. Hepatic LRP1 expression was significantly reduced in AAV8 miR-LRP1 mice compared with controls. Brain LRP1 expression was not significantly different between groups. Full gel images in Supplemental Fig. 3. **(C)** Representative hematoxylin and eosin (H&E)-stained liver sections from AAV8 Control and AAV8 miR-LRP1 mice showing no overt histopathological differences. Scale bar = 200 μm. **(D)** Serum alanine aminotransferase (ALT) levels in AAV8 Control and AAV8 miR-LRP1 mice, indicating no significant hepatotoxicity following AAV8 delivery. **(E)** Plasma cytokine profile showing no significant difference between AAV8 Control and AAV8 miR-LRP1 mice. TNF-α: tumor necrosis factor alpha, IL: interleukin, KC/GRO: keratinocyte chemoattractant (KC)/growth-regulated oncogene (GRO). Data are presented as mean ± SEM. Statistical comparisons were performed using an unpaired Student’s t-test. ns = not significant; ****p < 0.0001.

### 3.2. Effects of sustained hepato-specific LRP1 silencing on brain Aβ in APP/PS1 mice.

The effect of sustained hepato-specific LRP1 silencing on Aβ pathology in the APP/PS1 mouse brains was assessed by ELISA and histology. Previously, we demonstrated that hepato-specific silencing of LRP1 for 12 weeks increased soluble hAβ levels without affecting insoluble (Gu-HCl-soluble) hAβ and plasma hAβ levels [4]. Extending the duration of hepato-specific LRP1 silencing to 28 weeks resulted in a shift in this profile, with a significant increase in insoluble brain hAβ_1−42_ (198 ± 24% of control equivalent to 315 ± 27 ng/mg brain protein in the hepato-specific LRP1 silenced mice vs. 100 ± 25.1% of control equivalent to 113 ± 28 ng/mg brain protein in the AAV8-control mice, p < 0.05) (Fig. 2A) and plasma hAβ_1−42_ (164 ± 26% of control equivalent to 937 ± 146 pg/mL in the hepato-specific LRP1 silenced APP/PS1 mice vs. 100 ± 14.6% of control equivalent to 573 ± 84 pg/mL in the AAV8-control APP/PS1 mice, p < 0.05) (Fig. 2C). A similar trend of increased TBS-soluble brain hAβ_1−42_ was observed in hepato-specific LRP1 silenced APP/PS1 mice compared with the AAV8-control group (130 ± 6% of control equivalent to 1140 ± 53 pg/mg brain protein in the hepato-specific LRP1 silenced mice vs. 100 ± 15% of control equivalent to 878 ± 131 pg/mg brain protein in the AAV8-control mice, p = 0.094) (Fig. 2B), although this did not reach statistical significance. Further, plasma hAβ_1−42_ levels were strongly positively correlated with brain insoluble hAβ_1−42_ levels (r = 0.65, p < 0.05) (Fig. 2D), and no correlation was observed between plasma hAβ_1−42_ and soluble brain hAβ_1−42_ (Fig. 2E). Notably, hAβ_1−42_ levels in the liver were not detectable, possibly because of degradation and excretion in bile. In addition to hAβ_1−42_, which is derived from the overexpressed human APP in the APP/PS1 mice [22], we also measured mAβ_1−42_, which originates from endogenous mouse APP. No significant changes were observed in the levels of soluble and insoluble mAβ_1−42_, as well as plasma mAβ_1−42_ (Fig. 2F–H) between the hepato-specific LRP1-silenced APP/PS1 mice and the AAV8-control group.

The increased brain hAβ load with prolonged hepato-specific LRP1 silencing in APP/PS1 mice was confirmed using 6E10 immunostaining, which stains all isoforms of hAβ, including the precursor form [14]. Sustained hepato-specific LRP1 silencing in the APP/PS1 mice significantly increased the 6E10-positive Aβ count by 158% (p < 0.01) (Fig. 3A) and 6E10-positive Aβ area by 172% (p < 0.0001) (Fig. 3B), with no change in the average 6E10-positive Aβ stain size (Fig. 3C) compared with the AAV8 control group.

Representative 6E10-stained sagittal brain sections from hepato-specific LRP1-silenced and AAV8-control APP/PS1 mice are shown in Fig. 3D.

**(A)** Insoluble hAβ1–42 (Gu-HCl-soluble) levels in mouse brain were significantly elevated in AAV8 miR-LRP1 mice compared to AAV8 Control mice. **(B)** TBS-soluble human Aβ1–42 (hAβ1–42) levels in mouse brain homogenates trended toward an increase in AAV8 miR-LRP1 mice compared to AAV8 Control mice. **(C)** Circulating plasma hAβ1–42 levels were significantly increased in AAV8 miR-LRP1 mice relative to AAV8 Control mice. **(D)** Pearson correlation analysis revealed a significant positive correlation between brain insoluble and plasma hAβ1–42 (r = 0.65, p < 0.05). **(E)** No significant correlation was observed between brain soluble and plasma hAβ1–42 (r = 0.17, p = ns). **(F)** TBS-soluble and **(G)** insoluble (Gu-HCl-soluble) levels of endogenous mouse Aβ1–42 (mAβ1–42) in brain homogenates were not significantly different between AAV8 miR-LRP1 and AAV8 Control mice. **(H)** Circulating plasma mAβ1–42 levels showed no significant difference between AAV8 miR-LRP1 and AAV8 Control mice. Data are presented as mean ± SEM in A-C and F-H. Statistical comparisons in A-C and F-H were performed using unpaired Student’s t-tests, and using Pearson’s correlation in D-E. ns = not significant; *p < 0.05.

#### 1. Effect of hepato-specific LRP1 silencing on mature β-sheet rich amyloid plaque accumulation in aged APP/PS1 mice.

The effect of sustained hepato-specific LRP1 silencing on the mature β-sheet-rich Aβ plaque accumulation in the APP/PS1 mouse brain was assessed using Thioflavin-S staining. There was a significant increase in thioflavin-S positive area (p < 0.01, Fig. 4A) and count (p < 0.01, Fig. 4B), with no change in thioflavin-S average stain size (Fig. 4C) in the AAV8 LRP1 silenced APP/PS1 compared with AAV8-control APP/PS1 mice. Representative sagittal brain section images of thioflavin-S stains from the AAV8 control and AAV8 LRP1-silenced group are shown in Fig. 4D.

Quantification of 6E10-positive stain **(A)** number, **(B)** area, and **(C)** size in AAV8 miR-LRP1 and AAV8 Control mice. Significant increase in 6E10-positive stain number and area with hepatic LRP1 silencing. **(D)** Representative immunofluorescence images of sagittal brain sections stained with 6E10 antibody (green) in AAV8 Control (left) and AAV8 miR-LRP1 (right) mice. Scale bar = 400 μm. Data are presented as mean ± SEM of individual images analyzed from the AAV8 Control and AAV8 miR-LRP1 mice using the LME model with treatment as the fixed effect and mouse as the random effect. ns = not significant; **p < 0.01; ****p < 0.0001.

### 3.4. Effect of sustained hepato-specific LRP1 silencing on neuroinflammation in aged APP/PS1 mice.

Our previous work with short-term (12-week) hepato-specific-LRP1-silencing in APP/PS1 mice showed no change in microgliosis [4]. Sustained 28-week hepato-specific LRP1-silencing in APP/PS1 mice significantly increased GFAP (p < 0.01, Fig. 5A, D), TREM2 (p < 0.05, Fig. 5B, D), and ApoE (p < 0.05, Fig. 5E–F) immunoreactivity in AAV8-LRP1-silenced mouse brains compared with AAV8 controls. In contrast, a decreasing trend in Iba1-positive immunoreactive area was observed in AAV8-LRP1-silenced mouse brains compared with AAV8-controls (p = 0.068, Fig. 5C, D). Further, ApoE-positive area was strongly positively correlated with 6E10-positive Aβ area in the brain (r = 0.78, p < 0.0001, Fig. 5G).

Quantification of thioflavinS-positive stain **(A)** area, **(B)** number, and **(C)** size in AAV8 miR-LRP1 and AAV8 Control mice. Significant increase in thioflavinS-positive stain area and number with hepatic LRP1 silencing. **(D)** Representative images of sagittal brain sections stained with thioflavinS (green) in AAV8 Control (left) and AAV8 miR-LRP1 (right) mice. Scale bar = 400 μm. Data are presented as mean ± SEM of individual images analyzed from the AAV8 Control and AAV8 miR-LRP1 mice using the LME model with treatment as the fixed effect and mouse as the random effect. ns = not significant; **p < 0.01.

### 3.5. Behavior changes in APP/PS1 mice with prolonged hepato-specific LRP1 silencing.

Our previous work with short-term (12-week) hepato-specific LRP1-silencing in APP/PS1 mice showed increased hyperactivity but no change in spatial memory [4]. Consistent with this, prolonged hepato-specific LRP1 silencing in APP/PS1 mice showed a significant increase in the total distance traveled (p < 0.05, Fig. 6A, C) and mean speed (p < 0.05, Fig. 6B, C) during the open-field test compared with the AAV8-control-treated APP/PS1 mice. Notably, the magnitude of locomotion hyperactivity was greater with the 28-week hepatic-LRP1 silencing herein compared with the 12-week hepatic-LRP1 silencing we previously observed [4]. In addition, sustained hepato-specific LRP1 silencing in APP/PS1 mice resulted in a strong trend toward a reduction in the discrimination index for entries in the novel arm (p = 0.056, Fig. 6D–E) during the Y-maze test compared to the AAV8-control injected APP/PS1 mice, indicating spatial reference memory impairment. Finally, there was no significant change in the nesting behavior pattern between the AAV8-control and liver-specific LRP1-silenced groups (Fig. 6F).

#### 1. Liver-specific LRP1 silencing does not alter other Aβ uptake receptors or AD-relevant proteins but modulates APP levels in the liver

To determine whether the increased brain Aβ burden observed following liver-specific LRP1 silencing was due to alterations in other hepatic Aβ transport receptors, we assessed the expression of RAGE and MDR1, both of which have been implicated in Aβ transport [33, 52]. Immunoblot analysis demonstrated that hepatic LRP1 silencing did not significantly alter the expression of these receptors at 12 and 28 weeks (Fig. 7A–D). As shown in Fig. 7E, hepatocyte-specific LRP1 silencing resulted in a significant increase in hepatic APP levels at 12 weeks post-silencing, which normalized by 28 weeks (Fig. 7E). These findings suggest a temporal role for LRP1 in regulating hepatic APP expression. We next examined whether hepato-specific silencing of LRP1 affected other hepatic proteins associated with AD. As shown in Fig. 7F–I, no changes were observed in hepatic levels of ApoE, LRP6, or PSEN2. Additionally, plasma ApoE levels were unchanged following liver-specific LRP1 silencing (Fig. 7J). However, hepato-specific LRP1 silencing increased plasma levels of apolipoprotein B48 (ApoB48) (p < 0.05, Fig. 7K), the major apolipoprotein found in chylomicrons produced by the small intestine [23]. In contrast, plasma ApoB100 remained unchanged following liver-specific LRP1 silencing (Supplemental Fig. 2A).

**(A)** Representative western blots of liver tissue from AAV8 Control and AAV8 miR-LRP1 treated animals at 12 and 28 weeks. Densitometric analysis showing no change in hepatic **(B)** RAGE and **(C)** MDR1 expression, with a decrease in **(D)** hepatic LRP1 following LRP1 silencing at 12 and 28 weeks. **(E)** Representative western blots and densitometric quantification of hepatic APP expression after 12 and 28 weeks of hepatic LRP1 silencing. AAV8 miR-LRP1 treatment significantly increased liver APP at 12 weeks but not at 28 weeks. **(F)** Representative western blots of hepatic ApoE, LRP6, and PSEN2, in AAV8 Control and AAV8 miR-LRP1 mice. Densitometric quantification of **(G)** ApoE, **(H)** LRP6, and **(I)** PSEN2 hepatic expression, expressed as percentage of control; no significant differences were observed between groups. **(J)** Plasma ApoE levels showed no significant change between groups. **(K)** Plasma ApoB48 was significantly elevated in AAV8 miR-LRP1 mice. Full gel images in Supplemental Fig. 4. Data are expressed as mean ± SEM. Statistical comparisons were performed using unpaired t-tests. ns = not significant; *p < 0.05, ** p < 0.01.

## Discussion

Liver dysfunction is increasingly implicated in AD pathogenesis [3, 27, 31, 37], and identifying the underlying mechanisms may reveal novel therapeutic targets and improve AD diagnosis criteria. Hepatic LRP1, the principal receptor mediating peripheral Aβ uptake and degradation [33, 44, 45], is downregulated in liver disease [16, 19]; therefore, its loss is a potential mechanism by which liver pathology may impair peripheral Aβ clearance and promote cerebral Aβ accumulation [34]. Accordingly, individuals with the LRP1 rs1799986 T allele have higher LRP1 expression and reduced plasma Aβ levels [25, 39]. Building on our previous findings that 12 weeks of hepato-specific LRP1 silencing in APP/PS1 mice increased soluble brain Aβ and locomotor hyperactivity without affecting insoluble Aβ, neuroinflammation, or cognition [4], we extended the silencing period to 28 weeks to better model chronic hepatic LRP1 downregulation and determine whether prolonged loss drives a more severe AD phenotype. Our work shows that prolonged hepatic LRP1 silencing shifts the Aβ equilibrium toward insoluble Aβ accumulation, neuroinflammation, and cognitive deficits, underscoring hepatic LRP1 as a key regulator of brain Aβ homeostasis.

Hepato-specific LRP1 silencing was achieved by packaging LRP1-specific microRNA into an AAV8 vector under the control of the liver-specific TBG promoter [4]. This silencing approach demonstrated strong liver specificity and a favorable safety profile (Fig. 1). A 70% reduction in liver LRP1 expression, with unchanged LRP1 expression in the brain and kidney, preserved liver histology, normal ALT levels, and stable cytokine profiles, confirm no off-target effects or systemic inflammation with prolonged hepatic LRP1 silencing in our study.

The most significant finding of this study is the time-dependent progression of brain Aβ accumulation following hepatic LRP1 silencing. Together, our 12-week [4] and 28-week data (current study) elucidate the temporal dynamics of brain Aβ deposition in response to hepatic LRP1 modulation (Fig. 2–4). Our previous 12-week study showed an increase in soluble brain hAβ without affecting insoluble hAβ [4], suggesting that early hepatic LRP1 loss impacts soluble Aβ pools without promoting its aggregation. The current 28-week data reveal a progression of brain Aβ accumulation from soluble to insoluble forms, concomitant with an increase in plasma hAβ_1–42_. The total (6E10-positive) and mature (thioflavin-s-positive) Aβ plaque load were also significantly elevated with 28-week hepatic LRP1 silencing compared with controls. Notably, the magnitude of Aβ load increase was greater with 28-week (173%) than with 12-week (133%) silencing [4]. Therefore, prolonging hepatic LRP1 silencing from 12 to 28 weeks progressively exacerbates brain amyloidosis, consistent with a temporal profile in which initial soluble Aβ accumulation in the brain, driven by hepatic LRP1 loss, eventually crosses a critical threshold, which promotes insoluble Aβ deposition. This is further supported by a strong positive correlation between plasma hAβ_1–42_ and insoluble brain hAβ_1–42_ (r = 0.69, p < 0.05), and not between plasma hAβ_1–42_ and soluble brain hAβ_1–42_, twenty-eight weeks after silencing. These findings are consistent with Cheng et al., who also reported a significant increase in insoluble brain Aβ load following a comparable duration of hepatic LRP1 silencing (6 months) in APP/PS1 female mice [7]. Overall, our findings support a model in which hepatic LRP1-mediated peripheral Aβ clearance, when impaired, increases circulating Aβ levels and drives increased brain Aβ influx. Over time, this accumulation shifts from soluble to insoluble Aβ species, accelerating AD pathology.

The progressive increase in brain Aβ load following 28-week hepatic LRP1 silencing results in pronounced neuroinflammatory changes (Fig. 5). After 12 weeks of hepatic LRP1 silencing, lower Aβ levels coincided with no detectable changes in Iba1 expression [4], a marker of microglia and macrophage activation [20], or GFAP, a canonical marker of reactive astrogliosis [18] (unpublished data). This indicates that significant glial activation does not precede Aβ accumulation at this stage. After 28 weeks of hepatic LRP1 silencing, robust Aβ deposition was accompanied by marked upregulation of GFAP, along with a trend toward a reduction in Iba1 immunoreactivity but an increase in microglial TREM2 expression. Microglial TREM2 is a surface receptor that mediates phagocytosis, lipid sensing, and survival signaling [47]. Its upregulation is well-documented in response to increasing Aβ burden in AD [15], and the rise in TREM2 observed in the present study is consistent with a phenotypic transition from a homeostatic to a disease-associated microglial (DAM) state, likely driven by the progressive Aβ accumulation following 28 weeks of hepatic LRP1 silencing [26]. Additionally, the trend toward reduced Iba1 immunoreactivity with hepatic LRP1 silencing may indicate reduced microglial coverage, which could further contribute to Aβ plaque expansion [55]. Notably, the expression of brain ApoE, an apolipoprotein highly expressed by astrocytes [2], was significantly increased and positively correlated with Aβ load. In the AD brain, ApoE is closely associated with Aβ plaques, where it is known to play a role in both increasing Aβ aggregation into compact plaques and Aβ clearance [11, 36, 43, 51]. Interestingly, astrocyte-derived ApoE has been shown to reduce soluble Aβ but increase fibrillar Aβ plaques, accompanied by increased DAM [42]. Together, these findings suggest a plausible mechanistic sequence: prolonged hepatic LRP1 silencing drives Aβ accumulation, which in turn triggers astrogliosis and increased ApoE release. Elevated ApoE then promotes a shift from soluble to insoluble Aβ deposition, subsequently driving microglial transition toward a DAM phenotype, as evidenced by increased TREM2 expression.

Consistent with the progressive exacerbation of brain amyloidosis, prolonging hepatic LRP1 silencing from 12 to 28 weeks produced worsened behavioral outcomes (Fig. 6). The increase in locomotor hyperactivity, as measured by total distance traveled and mean speed in the open-field test, suggests that the duration of hepatic LRP1 silencing is positively associated with the severity of this behavioral phenotype, with the magnitude of hyperactivity being greater at 28 weeks than at 12 weeks [4]. Hyperactivity in APP/PS1 mice is associated with brain Aβ accumulation [50], and is frequently observed in AD, particularly as a non-cognitive behavioral symptom [10]. Importantly, the strong trend toward impaired spatial reference memory in the Y-maze test (p = 0.056), which was absent in the shorter 12-week study [4], suggests that progressive Aβ accumulation resulting from hepatic LRP1 silencing contributes to the gradual spatial reference memory deficits in APP/PS1 mice.

To investigate alternate mechanisms underlying increased brain Aβ burden, we first examined key hepatic Aβ-relevant receptors and APP processing pathways (Fig. 7). RAGE and MDR1 are key hepatic Aβ transporters that mediate Aβ uptake into the liver [33], and both represent potential compensatory receptors through which Aβ clearance could be altered following hepatic LRP1 loss. However, hepatic RAGE and MDR1 expression were unaffected by hepatic LRP1 silencing at 12 or 28 weeks, confirming hepatic LRP1 as the primary driver of the observed phenotype. Further, unchanged levels of hepatic ApoE, LRP6, and PSEN2 expression further support the conclusion that the observed brain and behavioral phenotypes are mediated through hepatic LRP1-specific pathways rather than a broad disruption of AD-relevant proteins.

The transient increase in hepatic APP at 12 weeks, which normalized by 28 weeks, is mechanistically intriguing (Fig. 7). Literature from the brain indicates that LRP1 can interact with APP at the cell surface and promote its internalization via endocytosis, after which APP is subsequently cleaved by secretases to generate Aβ, which is then released into the extracellular space [41, 46]; LRP1 can therefore increase Aβ levels in the brain [29, 54]. Accordingly, silencing or inhibition of LRP1 has been reported to decrease Aβ production and shift APP processing from amyloidogenic to non-amyloidogenic pathway [48]. Notably, the role of LRP1 in regulating APP processing in the liver has not been explored; however, based on the results of the current study, it is possible that hepatic LRP1 silencing reduces hepatic APP internalization, thereby increasing hepatic APP levels, which can be cleaved by secretases to increase peripheral Aβ levels. Notably, experimental evidence demonstrating that mice expressing hepatic human APP exhibit elevated peripheral Aβ and AD-related neurodegeneration provides causal support for hepatic APP as a contributor to AD pathology [30]. Taken together, hepatic LRP1 silencing may simultaneously impair peripheral Aβ clearance and transiently increase hepatic APP, creating a dual mechanism that increases plasma Aβ levels and drives brain Aβ accumulation.

Since the majority of circulating Aβ is bound to albumin (90%) and apolipoproteins (5%) [1], we measured their levels in plasma. Plasma albumin was slightly but significantly reduced with hepatic LRP1 silencing (Supplemental Fig. 2), which can diminish the sequestration of circulating Aβ, and contribute to elevated plasma and brain Aβ levels. Concurrently, hepatic LRP1 silencing increased plasma ApoB48, the structural apolipoprotein of intestinally derived chylomicron remnants [35], which is consistent with LRP1’s established role as a clearance receptor for ApoB48-containing remnant particles [49]. This is directly relevant to Aβ clearance, as the increased ApoB48 load can compete with Aβ for the residual 30% hepatic LRP1 expression (remaining after silencing), further reducing circulating Aβ clearance and driving brain Aβ accumulation. An additional mechanism underlying ApoB-mediated brain Aβ increase is impairment of the blood-brain barrier (BBB) [30]. However, in our study, we did not observe increases in brain IgG and brain ApoB levels (Supplemental Fig. 2), indicating the absence of large BBB disruptions and plasma ApoB migration into the brain. Finally, we measured ApoB100 and ApoE, and given their reduced reliance on LRP1 or reliance on both the LDL receptor and LRP1 for clearance [13, 49], no change in their levels was observed with hepatic LRP1 silencing.

The study has some limitations. First, the study did not evaluate sex-specific effects of hepatic LRP1 silencing on AD pathology. Given that hepatic LRP1 silencing resulted in a similar increase in brain amyloidosis in female mice [7], we expect that a similar progressive worsening of AD pathology may be observed in female APP/PS1 mice. Second, we did not study the impact of hepatic LRP1 silencing on tauopathy, which is important given that tau pathology more closely predicts cognitive decline in AD [21]. Third, the role of hepatic LRP1 in regulating APP processing in the liver is largely unexplored and warrants further investigation.

## Conclusions

Sustained hepatic LRP1 loss drives progressive AD pathology through increased peripheral Aβ, disrupted plasma ApoB48 levels, and transiently increased hepatic APP expression, the latter suggesting a dual mechanism in which reduced Aβ clearance and increased peripheral Aβ production via hepatic APP jointly increase plasma Aβ levels. Together, these mechanisms shift the brain Aβ equilibrium from soluble to insoluble species over time, culminating in Aβ plaque deposition, neuroinflammation, and worsening behavioral deficits. The absence of compensatory changes in other hepatic Aβ transporters confirms that these effects are driven by hepatic LRP1. Future work should determine whether restoring hepatic LRP1 function can reduce AD pathology in models of liver injury and whether it represents a viable peripheral biomarker for AD risk in liver disease.

## Supplementary Material

Supplementary Files

This is a list of supplementary files associated with this preprint. Click to download.

- SupplementaryfileChandrashekarRoulesetal.docx
- Graphicalabstract.jpeg

## Figures and Tables

**Figure 1:**
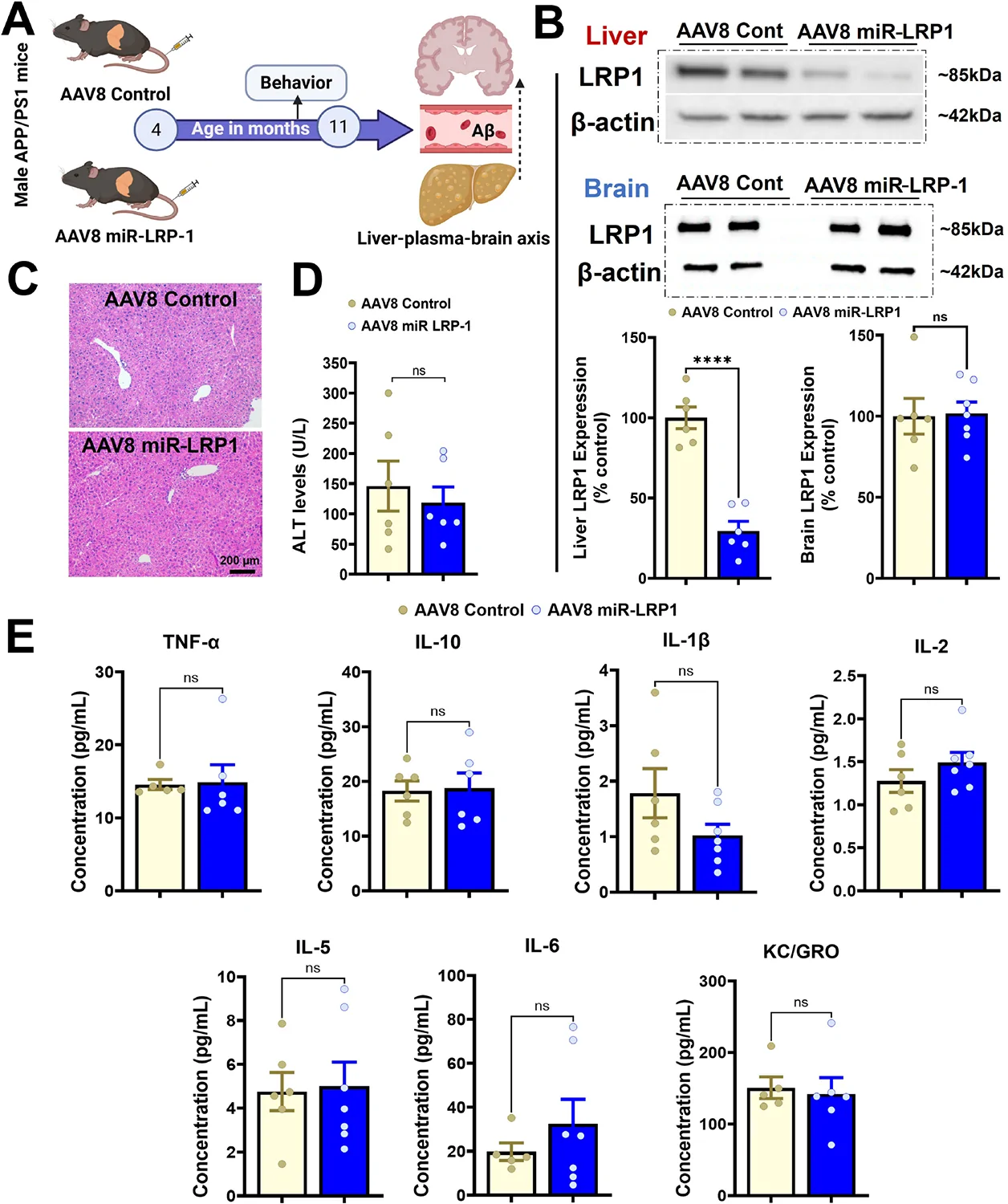
Liver-specific silencing of LRP1 via AAV8-mediated miRNA delivery does not affect brain LRP1 expression, hepatic integrity, or peripheral inflammation. **(A)** Schematic of the experimental design created using BioRender. Male APP/PS1 mice were injected with either AAV8 Control or AAV8 miR-LRP1 at 4 months of age and aged to 11 months. Animals underwent a battery of behavioral assessments, including nesting, open-field, and Y-maze tests. At endpoint, brain, plasma, and liver tissues were collected to assess amyloid-beta (Aβ) levels and LRP1 expression. **(B)**Representative western blots of LRP1 protein expression in liver (top, red panel) and brain (middle, blue panel) from AAV8 Control and AAV8 miR-LRP1 mice. Hepatic LRP1 expression was significantly reduced in AAV8 miR-LRP1 mice compared with controls. Brain LRP1 expression was not significantly different between groups. Full gel images in Supplemental Figure 3. **(C)**Representative hematoxylin and eosin (H&E)-stained liver sections from AAV8 Control and AAV8 miR-LRP1 mice showing no overt histopathological differences. Scale bar = 200 μm. **(D)** Serum alanine aminotransferase (ALT) levels in AAV8 Control and AAV8 miR-LRP1 mice, indicating no significant hepatotoxicity following AAV8 delivery. **(E)** Plasma cytokine profile showing no significant difference between AAV8 Control and AAV8 miR-LRP1 mice. TNF-α: tumor necrosis factor alpha, IL: interleukin, KC/GRO: keratinocyte chemoattractant (KC)/growth-regulated oncogene (GRO). Data are presented as mean ± SEM. Statistical comparisons were performed using an unpaired Student’s t-test. ns = not significant; ****p < 0.0001.

**Figure 2:**
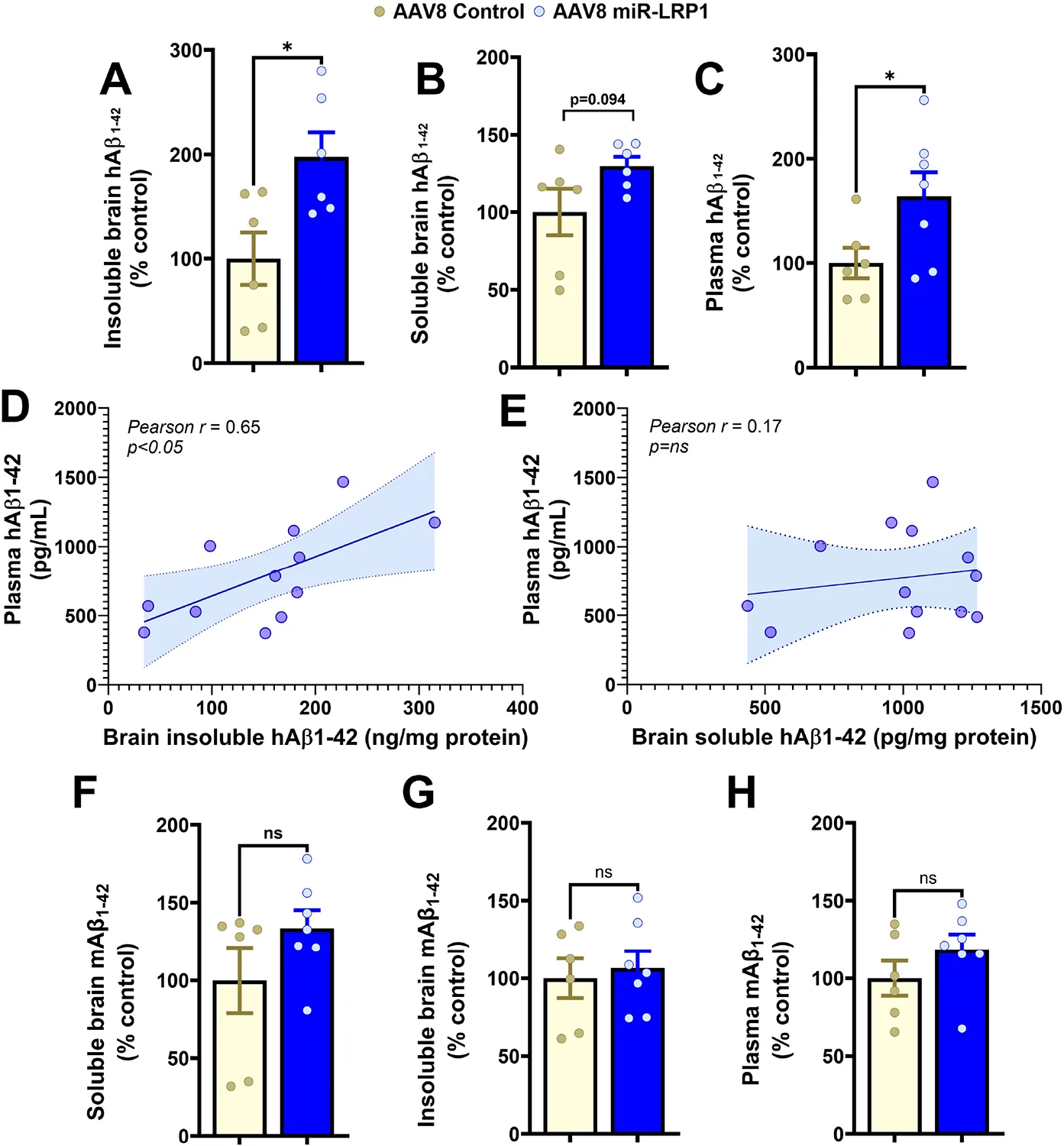
Liver-specific LRP1 silencing increases insoluble brain and plasma human Aβ42 with no change in mouse Aβ42 levels in APP/PS1 mouse brains. **(A)** Insoluble hAβ1–42 (Gu-HCl-soluble) levels in mouse brain were significantly elevated in AAV8 miR-LRP1 mice compared to AAV8 Control mice. **(B)** TBS-soluble human Aβ1–42 (hAβ1–42) levels in mouse brain homogenates trended toward an increase in AAV8 miR-LRP1 mice compared to AAV8 Control mice. **(C)** Circulating plasma hAβ1–42 levels were significantly increased in AAV8 miR-LRP1 mice relative to AAV8 Control mice. **(D)** Pearson correlation analysis revealed a significant positive correlation between brain insoluble and plasma hAβ1–42 (r = 0.65, p < 0.05). **(E)** No significant correlation was observed between brain soluble and plasma hAβ1–42 (r = 0.17, p = ns). **(F)** TBS-soluble and **(G)** insoluble (Gu-HCl-soluble) levels of endogenous mouse Aβ1–42 (mAβ1–42) in brain homogenates were not significantly different between AAV8 miR-LRP1 and AAV8 Control mice. **(H)** Circulating plasma mAβ1–42 levels showed no significant difference between AAV8 miR-LRP1 and AAV8 Control mice. Data are presented as mean ± SEM in A-C and F-H. Statistical comparisons in A-C and F-H were performed using unpaired Student’s t-tests, and using Pearson’s correlation in D-E. ns = not significant; *p < 0.05.

**Figure 3:**
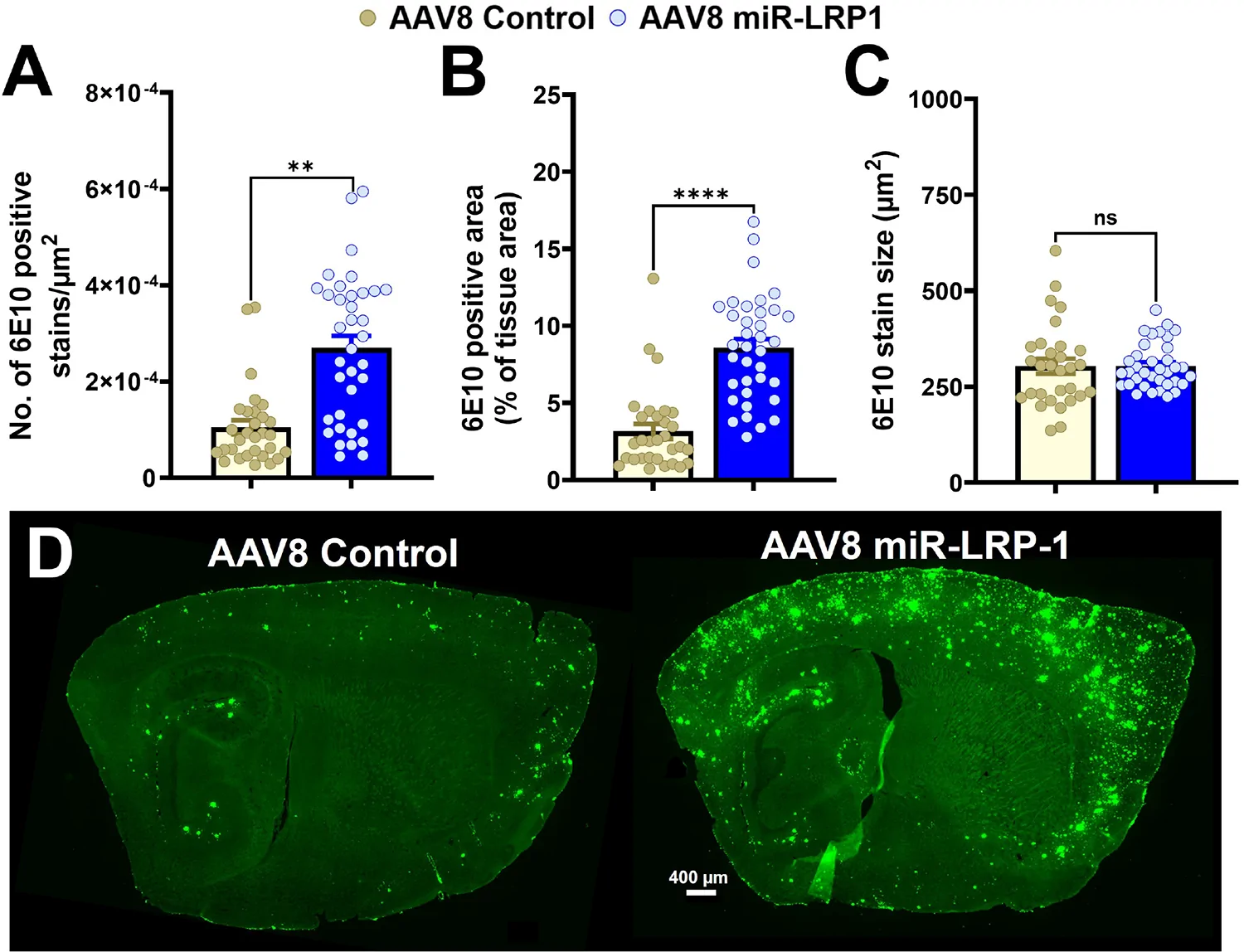
Immunostaining confirms increased human Aβ burden in APP/PS1 mouse brains following liver-specific LRP1 silencing. Quantification of 6E10-positive stain **(A)**number, **(B)** area, and **(C)** size in AAV8 miR-LRP1 and AAV8 Control mice. Significant increase in 6E10-positive stain number and area with hepatic LRP1 silencing. **(D)** Representative immunofluorescence images of sagittal brain sections stained with 6E10 antibody (green) in AAV8 Control (left) and AAV8 miR-LRP1 (right) mice. Scale bar = 400 μm. Data are presented as mean ± SEM of individual images analyzed from the AAV8 Control and AAV8 miR-LRP1 mice using the LME model with treatment as the fixed effect and mouse as the random effect. ns = not significant; **p < 0.01; ****p < 0.0001.

**Figure 4:**
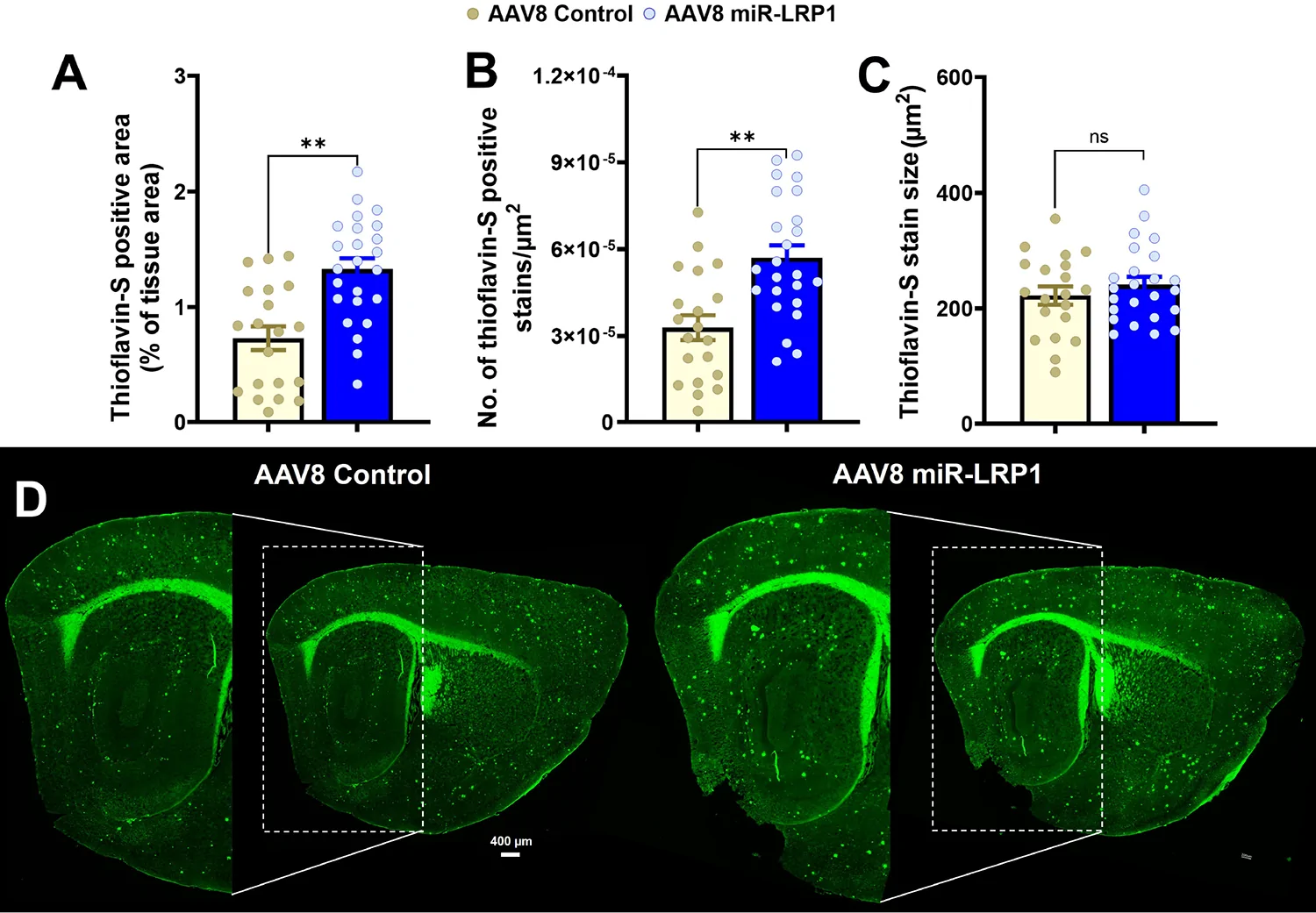
Liver-specific LRP1 silencing increases mature Aβ plaques in APP/PS1 mouse brains. Quantification of thioflavinS-positive stain **(A)** area, **(B)** number, and **(C)** size in AAV8 miR-LRP1 and AAV8 Control mice. Significant increase in thioflavinS-positive stain area and number with hepatic LRP1 silencing. **(D)** Representative images of sagittal brain sections stained with thioflavinS (green) in AAV8 Control (left) and AAV8 miR-LRP1 (right) mice. Scale bar = 400 μm. Data are presented as mean ± SEM of individual images analyzed from the AAV8 Control and AAV8 miR-LRP1 mice using the LME model with treatment as the fixed effect and mouse as the random effect. ns = not significant; **p < 0.01.

**Figure 5:**
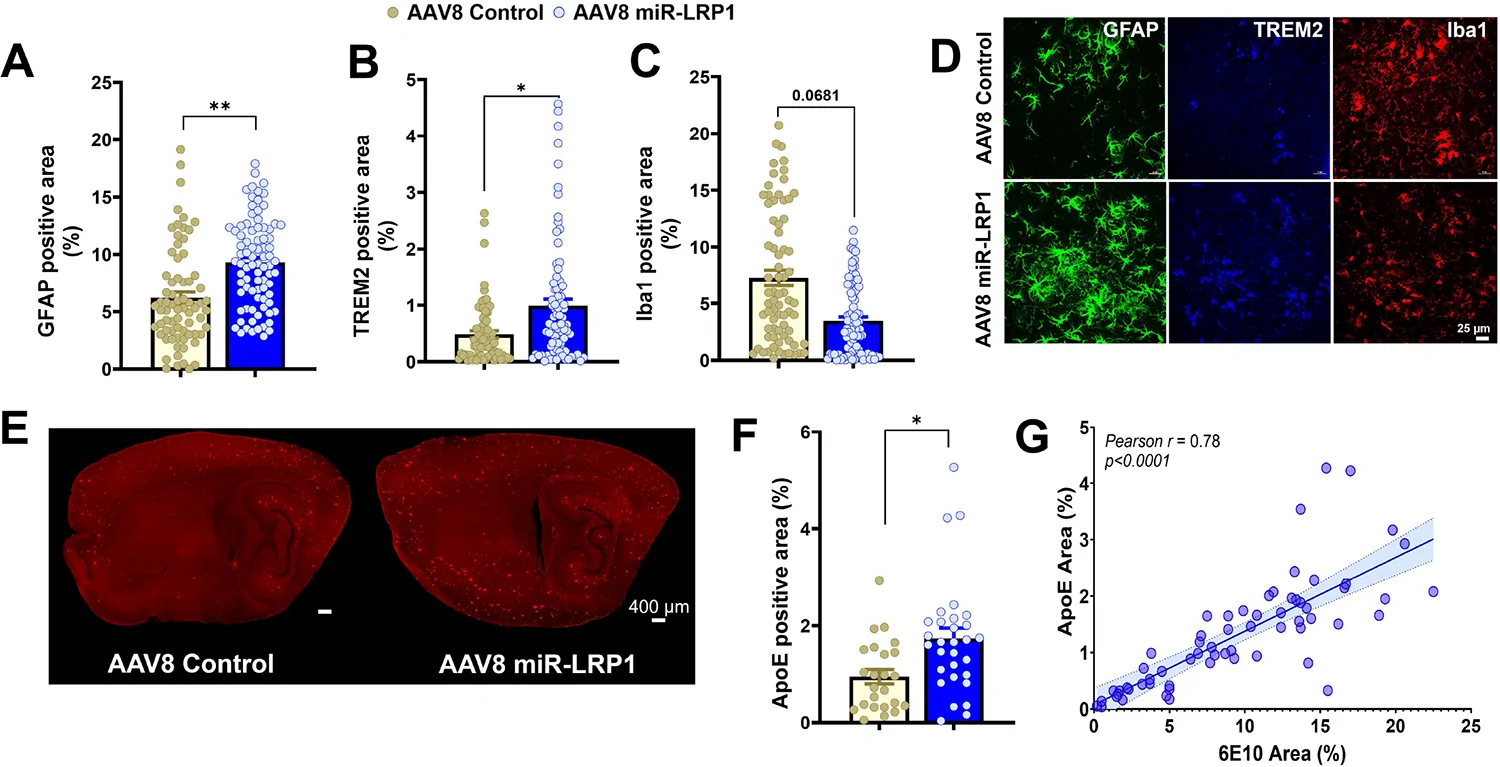
Significant alteration in neuroinflammation with liver-specific LRP1 silencing in APP/PS1 mice. A significant increase in **(A)**GFAP-positive area and **(B)** TREM2-positive area, and **(C)** a decreased trend for Iba1-positive area in AAV8 miR-LRP1 compared with AAV8 Control mice. **(D)** Representative immunofluorescence images showing GFAP (green), TREM2 (blue), and Iba1 (red). Scale bar = 25 μm. **(E)**Representative immunofluorescence images showing ApoE (red). Scale bar = 400 μm. **(F)** A significant increase in brain ApoE immunoreactivity in AAV8 miR-LRP1 compared with AAV8 Control mice. **(G)** Pearson correlation analysis showed that ApoE immunoreactive area was positively correlated with 6E10-positive area. Data are presented as mean ± SEM of individual images analyzed from the AAV8 Control and AAV8 miR-LRP1 mice using the LME model with treatment as the fixed effect and mouse as the random effect in A-C and F. ns = not significant; * p < 0.05, **p < 0.01.

**Figure 6:**
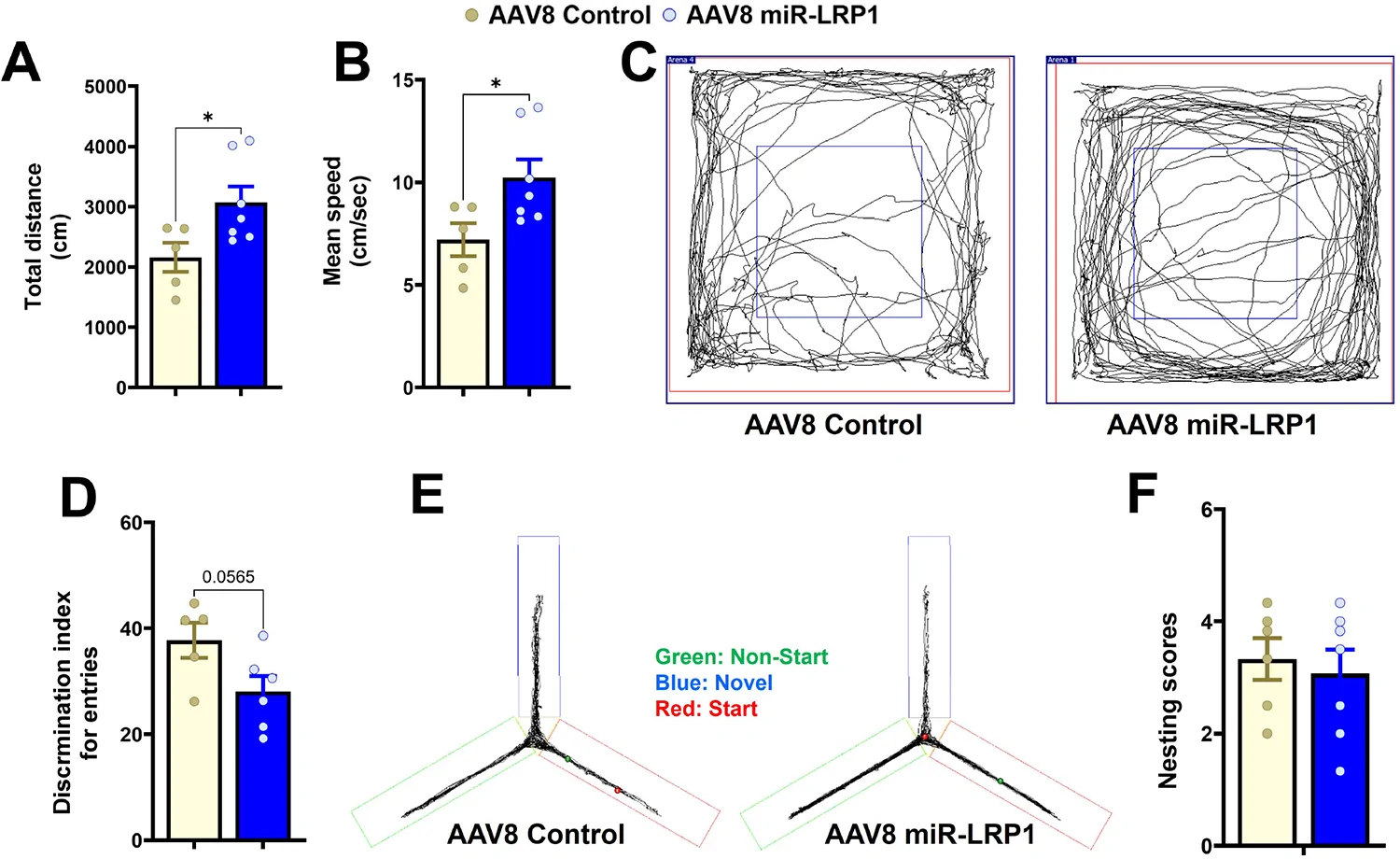
Significant behavior changes with prolonged liver-specific LRP1 silencing in APP/PS1 mice. In open-field analysis, there was a significant increase in the **(A)** total distance traveled and **(B)**mean speed in AAV8 miR-LRP1 mice compared with AAV8 Control mice. **(C)**Representative trajectory maps of the open-field test. **(D)**Discrimination index showed a strong decreasing trend in AAV8 miR-LRP1 mice compared with AAV8 Control mice. **(E)** Representative trajectory maps of the Y-maze test. **(F)** There was no significant difference in nesting scores in AAV8 miR-LRP1 mice compared to the control group. Data are presented as Mean ± SEM and were analyzed using unpaired Student’s t-test or the Mann-Whitney U test. *p < 0.05.

**Figure 7:**
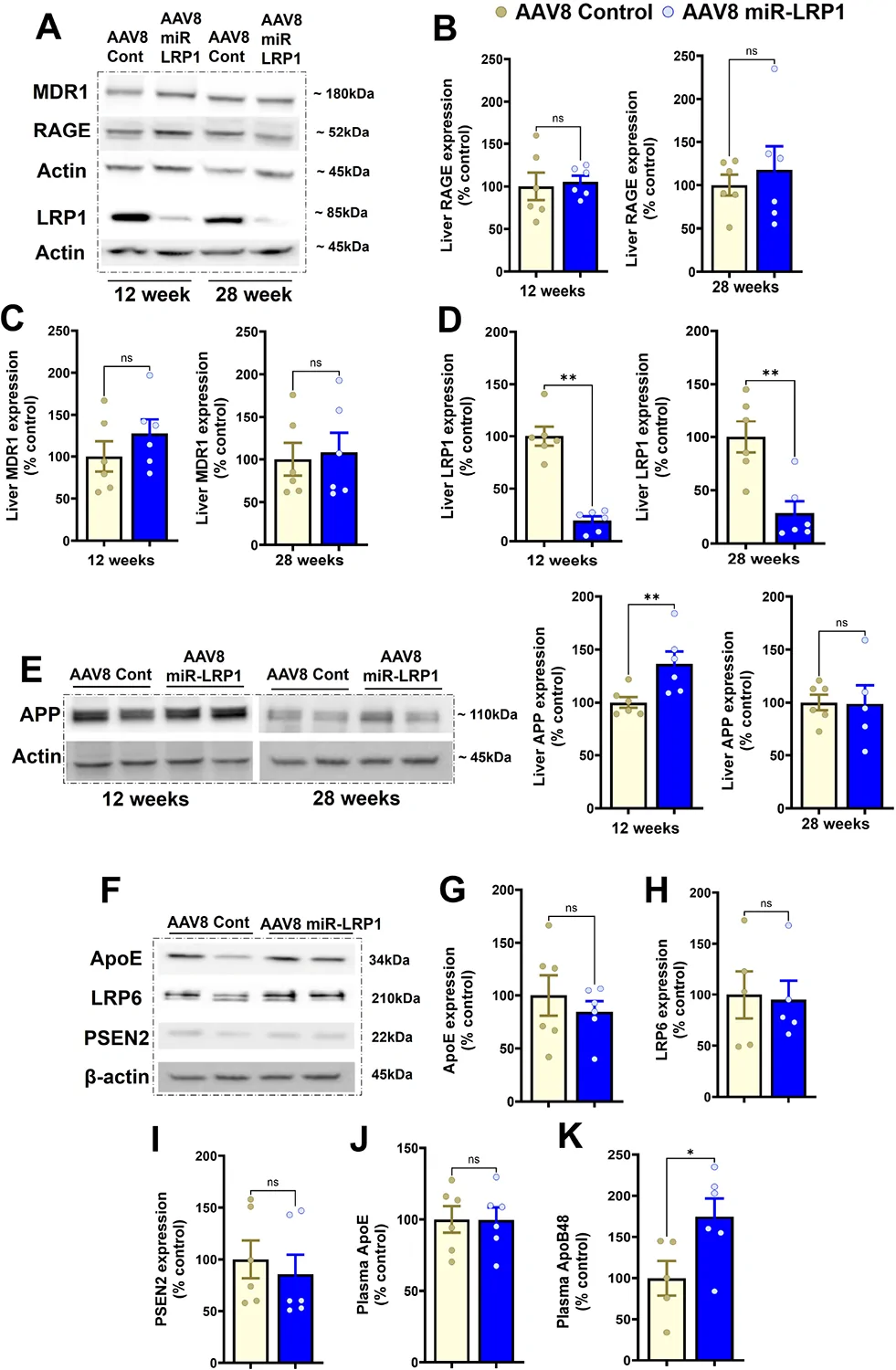
Effects of liver-specific LRP1 silencing on amyloid-related protein expression. **(A)** Representative western blots of liver tissue from AAV8 Control and AAV8 miR-LRP1 treated animals at 12 and 28 weeks. Densitometric analysis showing no change in hepatic **(B)** RAGE and **(C)**MDR1 expression, with a decrease in **(D)** hepatic LRP1 following LRP1 silencing at 12 and 28 weeks. **(E)** Representative western blots and densitometric quantification of hepatic APP expression after 12 and 28 weeks of hepatic LRP1 silencing. AAV8 miR-LRP1 treatment significantly increased liver APP at 12 weeks but not at 28 weeks. **(F)** Representative western blots of hepatic ApoE, LRP6, and PSEN2, in AAV8 Control and AAV8 miR-LRP1 mice. Densitometric quantification of **(G)** ApoE, **(H)** LRP6, and **(I)**PSEN2 hepatic expression, expressed as percentage of control; no significant differences were observed between groups. **(J)** Plasma ApoE levels showed no significant change between groups. **(K)** Plasma ApoB48 was significantly elevated in AAV8 miR-LRP1 mice. Full gel images in Supplemental Figure 4. Data are expressed as mean ± SEM. Statistical comparisons were performed using unpaired t-tests. ns = not significant; *p < 0.05, ** p < 0.01.

## Data Availability

All data supporting the findings of this study are available within the paper and its Supplementary Information.

## Abbreviations

AAV8: Adeno-associated virus 8
ALT: Alanine transaminase
ALP: Alkaline phosphatase
AD: Alzheimer’s disease
Aβ: Amyloid beta
APP: Amyloid precursor protein
BBB: Blood-brain barrier
BUN: Blood urea nitrogen
BSA: Bovine serum albumin
Gu-HCl: Guanidine hydrochloride
H&E: Hematoxylin/eosin
hAβ: Human Aβ
LDL: Low-density lipoprotein
LRP1: Low-density lipoprotein receptor-related protein 1
LME: Linear mixed effect
mAβ: Mouse Aβ
MDR1: Multidrug resistance protein 1
PBS: Phosphate-buffered saline
PVDF: Polyvinylidene fluoride
PSEN2: Presenilin 2
RIPA: Radioimmunoprecipitation assay buffer
RAGE: Receptor for advanced glycation end products
TBG: Thyroxine binding globulin
TBS: Tris-buffered saline
